
JOURNAL INFORMATION
==============================
NLM Title Abbreviation: Buffalo Med J
NLM Journal ID: 0027534

Buffalo Medical Journal

ISSN: 1040-3817
Publisher: Biomedical Journal Digitization Project

ARTICLE INFORMATION
==============================
PMCID: PMC8731554
PMID: 36886097
Article version: 1
Subjects: Literary Notes

Literary Notes

Print publication date: 1902 Aug
Volume: 42
Volume Series: New Ser
Issue: 1
First page: 76
Last page: 76
License: This work is free of known copyright restrictions. For more information, please see PMC Back Issue Digitization.
License URL: https://creativecommons.org/publicdomain/mark/1.0/

==============================

Medical and Surgical Report of the Presbyterian Hospital, New York. Volume V. January, 1902. Edited by Drs. Andrew J. McCosh and W. Gilman Thompson.

The Roller Bandage. By William Barton Hopkins, surgeon to Pennsylvania Hospital and to the Orthopedic Hospital and Infirmary for Nervous Diseases. 12mo, pp. 178. Fifth edition, revised. Philadelphia: J. B. Lippincott Company. 1902. [Price, $1 50.)

Vaughan and Novy on Cellular Toxins. A treatise on Cellular Toxins, or the Chemical Factors in the Causation of Disease. By Victor C. Vaughan, Ph.D., M.D.,Professor of Hygiene and Physiological Chemistry, and Frederick G. Novy, M. D., Junior Professor of Hygiene and Physiological Chemistry in the University of Michigan. New (4th) edition, revised and enlarged. In one 8vo volume of 480 pages, with six illustrations. Lea Brothers & Co., Philadelphia and New York. 1902. [$3.00 net.]

The Practical Medicine Series of Year Books. Ten volumes. Issued monthly under the general editorial charge of Gustavus P. Head, M. D., Professor of Laryngology and Rhinology in the Chicago Post-Graduate Medical School. Volume VII. Materia Medica and Therapeutics; Preventive Medicine; Climatology; Forensic Medicine. By George F. Butler, Ph.G., M.D., Henry B. Favill, A.B., M.D., Norman Bridge, A.B., M.D., Harold N. Moyer, M.D. 12mo, pp. 279. Chicago: The Year Book Publishers. 1902. [$1.50. Entire series, $7.50.]

The Johns Hopkins Hospital Reports. Volume X., Nos. 3, 4 and 5. Baltimore: The Johns Hopkins Press. 1902.

---

### LITERARY NOTES.

The *Medical Book News* is the title of a bimonthly brochure issued by P. Blakiston's Son & Company, of Philadelphia. It is devoted to the literature of medicine and allied sciences, and its object, as announced by the publishers, is to furnish information of use to physicians and others in selecting and purchasing such books of professional and scientific nature as they need. It will give descriptions of important books, reviews from medical magazines, news items, lists of the most recent American and English books of all publishers, and of new books on special subjects. The first number is dated July, 1902, and is a small octavo, beautifully printed in double columns. The publishers propose to send it without charge to all who are interested in its purposes, a liberality for which they certainly deserve all praise.

---

The *Old Dominion Journal of Medicine and Surgery* is the name of a new periodical published at Richmond and edited by Dr. Greer Baughman, assisted by Dr. A. B. Greiner. It is the official organ of the alumni society of the Medical College of Virginia. The college is the thirteenth oldest medical school in the United States and such a foundation ought to insure the successful career of the magazine, which we confidently bespeak.

---

WANTED—A young physician, recently graduated, desirous of occupation at once, can obtain a position at a Sanitarium where hot air treatments, electricity, baths, etc., are administered. Apply to G. S., 685 Linwood Avenue, Buffalo, N. Y.
